# Editorial: Thyroid cancer: New perspectives in diagnosis and therapy

**DOI:** 10.3389/fphar.2022.1057731

**Published:** 2022-10-31

**Authors:** Rhitajit Sarkar, Stephanie L. Lee, Viksit Kumar, Shilpa Thakur

**Affiliations:** ^1^ Laboratory of Endocrinology and Receptor Biology, National Institute of Diabetes and Digestive and Kidney Diseases, Bethesda, MD, United States; ^2^ Department of Medicine, Boston University School of Medicine, Boston, MA, United States; ^3^ Department of Radiology, Massachusetts General Hospital, Boston, MA, United States; ^4^ National Institutes of Health, Bethesda, MD, United States

**Keywords:** thyroid cancer (TC), thyroid surgery, thyroid nodule characterization, thyroid ultrasonography, thyroid diagnosis, targeted radiotherapeutics

With the advancement of high-throughput molecular technologies and associated interventional methods, our understanding of the molecular changes accompanying thyroid cancer (TC) initiation and progression have improved significantly. The addition of in-depth analytical methods for conventional techniques and analyses of novel biomarkers have resulted in remarkable enhancement in TC diagnosis, patient risk stratification, and identification of targets for precision therapy. Interestingly, profiling thyroid tumors and their risk of associated metastases has allowed the translation of newer findings into clinical use on a “bench-to-bedside” strategy. This Research Topic, “Thyroid cancer: New perspectives in diagnosis and therapy,” contains a collection of studies that address emerging questions in TC diagnosis and detailed approaches to find the relevant intervention to improve TC prognosis.

For decades, ultrasonography has been a convenient and straightforward imaging technique used to study the characteristics of TC, although incurring several limitations in determining the metastatic potential of primary TC. Xue et al. demonstrate the feasibility of contrast-enhanced ultrasound in diagnosing malignant thyroid nodules and predicting cervical lymph node metastasis. This study can improve the preoperative diagnosis of lymph node metastasis and help decide on individual lymph node dissection to enhance patients’ postoperative quality of life.

In keeping with this theme, the research article by Qi et al. addresses the role of multiple nomographic prediction models based on clinical, pathological, and US characteristics to predict the risk of Delphian lymph node metastasis in papillary thyroid carcinoma. Using multiple logistic regression models to build prediction models and draw interactive nomograms provides critical insight into the correlation of lymph node metastasis with the invasiveness of TC.

To complement the findings of ultrasound imaging and fine-needle aspiration techniques for preoperative evaluation of benign, malignant, or metastatic thyroid nodules, the discovery of non-invasive biomarkers is incumbent. The original research of Zhang et al. establishes the potential of IgG N-glycans as biomarkers to discriminate amongst TC patients from benign thyroid nodules and healthy individuals, enabling early-stage detection. Employing a high-throughput quantitative strategy using mass spectrometry to detect IgG glycan biomarkers justifies the ease of the analyses to be readily performed in a clinical setup.

Correlation of distant metastasis is vital to evaluate the risk factors associated with another variant of differentiated TC, *viz.*, follicular thyroid cancer (FTC), which is often challenging to manage due to its likelihood of metastasizing. The article by Wu et al. determines the impact of metastasis on cancer-specific survival while considering age as a significant risk factor for distant metastasis of FTC. The study also demonstrates that distant metastases’ sites varied whether diagnosed at presentation or developed during follow-up.

Finally, Luo et al. associated urinary iodine excretion with progression-free survival for patients with differentiated TC undergoing RAI therapy, showing that high iodine excretion may link with a worse prognosis. They also derive that the presence of distant metastases at diagnosis is a stronger independent predictor of progression–a finding that corroborated with several articles in this series. Combinedly, the collection of articles on this topic addresses novel, emerging approaches to thyroid cancer diagnosis and treatment.

